# Effectiveness of a Geographic Information System-Integrated Mobile Platform for Coordinating Early Stage Rehabilitation After Total Hip Arthroplasty: A Randomized Controlled Trial

**DOI:** 10.3390/ijerph23060751

**Published:** 2026-06-03

**Authors:** Zhandos Kurban, Sholpan Bulekbayeva, Natalia Slivkina, Elena Titskaya, Yersin Ussin, Galym Zorgulov, Farkhad Adylkhanov, Dana Aldakuatova

**Affiliations:** 1 Department of Rehabilitation and Sports Medicine, NCJSC Astana Medical University, Astana 010000, Kazakhstan; 2 National Scientific Center for the Development of the Social Protection Sector, Almaty 050000, Kazakhstan; 3 Laboratory for Planning and Development of Medical Technologies, Tomsk Research Institute of Balneology and Physiotherapy, Branch of the Federal Scientific and Clinical Center for Medical Rehabilitation and Balneology of the Federal Medical and Biological Agency, Tomsk 634000, Russia; 4 Green Clinic LLC, Astana 010000, Kazakhstan; 5 Scientific and Educational Center of Surgery, NCJSC Astana Medical University, Astana 010000, Kazakhstan; 6 Department of Epidemiology and Biostatistics, NCJSC Astana Medical University, Astana 010000, Kazakhstan

**Keywords:** total hip arthroplasty, rehabilitation, geographic information system, digital health, quality of life, health services accessibility

## Abstract

**Highlights:**

**Public health relevance—How does this work relate to a public health issue?**

- Post-operative rehabilitation after total hip arthroplasty (THA) is a critical but systematically underserved component of care, with patients in many healthcare systems—including Kazakhstan—facing multi-month waiting lists and information gaps that delay recovery and violate the principle of early rehabilitation initiation.
- Geographic fragmentation of rehabilitation services and informational asymmetry between patients, primary care physicians, and specialist facilities represent a structural public health challenge that digital coordination tools are uniquely positioned to address.

**Public health significance—Why is this work of significance to public health?**

- This randomized controlled trial demonstrates that a GIS-integrated mobile coordination platform significantly reduces time to rehabilitation initiation by 16 days and improves physical health-related quality of life and QALYs at 12 months compared to standard referral pathways.
- By providing patients and clinicians with transparent, real-time information on rehabilitation facility availability and enabling direct patient-to-provider matching, the platform offers a replicable approach to reducing care coordination failures and broadening equitable access to post-surgical rehabilitation.

**Public health implications—What are the key implications or messages for practitioners, policy makers and/or researchers in public health?**

- Surgical departments and health systems should consider integrating GIS-enabled coordination platforms into standard THA discharge protocols, as earlier rehabilitation initiation translates into measurable improvements in physical functioning and health utility at the population level.
- Health system administrators and researchers should evaluate the transferability of GIS-enabled coordination platforms to other rehabilitation contexts—including post-stroke, cardiac, and trauma rehabilitation—where similar barriers of informational asymmetry and unequal resource distribution persist.

**Abstract:**

Total hip arthroplasty (THA) is among the most effective orthopedic interventions for osteoarthritis, yet post-operative rehabilitation is frequently delayed due to informational and organizational barriers. Geographic information system (GIS) technology offers a promising approach to improving rehabilitation access coordination, though its integration into patient-facing mobile platforms remains insufficiently studied. This two-arm, parallel-group, superiority randomized controlled trial enrolled 142 adult patients (≥18 years) within seven days of primary THA at the National Research Oncology Center LLC, Astana, Kazakhstan. Participants were randomized 1:1 to the GIS-integrated Health-GIS mobile coordination platform (experimental group) or standard general practitioner (GP)-mediated referral (control group). Key exclusion criteria included severe cognitive or visual impairment, absence of smartphone access or digital literacy, and medical contraindications to rehabilitation. The primary outcomes were time to second-stage rehabilitation initiation and health-related quality of life assessed by the SF-12 (Physical and Mental Component Summaries). Secondary outcomes included the Harris Hip Score (HHS), Visual Analogue Scale (VAS) for pain, System Usability Scale (SUS), and quality-adjusted life years (QALYs) over a 12-month follow-up. Of 142 randomized participants (61% male, 39% female), 131 completed follow-up and were included in the modified intention-to-treat analysis (experimental: *n* = 66; control: *n* = 65). The experimental group initiated second-stage rehabilitation significantly earlier (median 43 vs. 59 days; *p* = 0.021). At 12 months, the experimental group demonstrated superior SF-12 Physical Component Summary scores (48.21 vs. 42.84; *p* < 0.001), while Mental Component Summary scores did not differ significantly between groups (46.96 vs. 47.05; *p* = 0.669). Quality-adjusted life years were significantly higher in the experimental group (0.74 ± 0.04 vs. 0.72 ± 0.04; *p* = 0.008). Harris Hip Scores were significantly better in the experimental group at 6 weeks (*p* < 0.001) and 6 months (*p* = 0.009), converging by 12 months (*p* = 0.068). No statistically significant between-group differences in pain intensity (VAS) were observed at any time point (baseline: *p* = 0.814; 6 weeks: *p* = 0.336; 6 months: *p* = 0.066; 12 months: *p* = 0.105). Platform usability was rated as good-to-excellent by clinicians (SUS: 86.9 at 6 months) and acceptable by patients (mean SUS: 71.4). A GIS-integrated mobile coordination platform significantly reduced time to rehabilitation initiation and improved physical health-related quality of life and health utility following THA compared to standard referral practice. These findings support platform-based care coordination as an effective complement to surgical care, with important implications for rehabilitation access policy. Future multi-center studies and formal cost-effectiveness analyses are warranted to establish generalizability. Trial Registration: ClinicalTrials.gov, NCT07201116, registered 23 September 2025.

## 1. Introduction

Total hip arthroplasty (THA) ranks among the most successful surgical interventions in contemporary orthopedics, consistently demonstrating high efficacy in restoring joint function and improving patients’ quality of life [1,2]. In 2019, approximately 528 million people worldwide were living with osteoarthritis, driving substantial and growing demand for joint replacement procedures [3,4]. This burden continues to escalate alongside population aging and the rising prevalence of degenerative joint disease [4]. Projections across developed nations point to dramatic increases in THA demand: in the United States, procedure volumes are forecast to rise 71% by 2030, reaching 635,000 annually [5]; in Australia, a 198% increase to 94,086 procedures is anticipated by 2046 [6]; and in Japan, projected procedure numbers for 2030 among individuals aged 65–74 stand at 9005 for men and 36,416 for women, representing increases of 147% and 99%, respectively, compared to 2018 [7]. Yet the success of surgical treatment depends critically on the quality and accessibility of post-operative rehabilitation, particularly during the early recovery period [8].

The prevailing framework of medical rehabilitation holds that early initiation, continuity, and care coordination produce superior clinical outcomes [9,10,11]. The early rehabilitation phase—spanning up to three to six months following surgery—is considered important for restoring full physical function, establishing correct movement patterns, and achieving social reintegration [12,13].

Physical therapy following THA constitutes a specialized, multi-component clinical discipline that extends substantially beyond the general concept of rehabilitation. It encompasses individualized therapeutic exercise aimed at restoring lower extremity strength and mobility, neuromuscular re-education, gait training and fall prevention, and progressive strengthening and cardiorespiratory reconditioning programs [8,12,13]. Each of these interventions requires repeated in-person patient assessment, hands-on correction of movement patterns, individualized adjustment of exercise intensity and loading, and continuous clinical monitoring by a qualified physical therapist [8,12]. Direct specialist supervision is of particular importance during the early postoperative period, when restoration of normal gait biomechanics, safe progressive loading of the prosthetic joint, and prevention of the establishment of compensatory movement strategies represent the primary rehabilitation priorities [8,12]. Notably, a systematic review with meta-analysis demonstrated that biomechanical gait abnormalities and compensatory movement patterns frequently persist following THA, particularly in patients with a prolonged history of osteoarthritis, underscoring the need for targeted corrective intervention under the direct in-person supervision of a physical therapist [14]. In this context, the use of GIS-integrated mobile platforms is conceived not as a substitute for in-person interaction with a physical therapist, but as an essential logistical bridge ensuring the most rapid possible patient access to inpatient physical therapy.

Despite well-established principles governing the organization of rehabilitation care, real-world practice reveals significant systemic failures across healthcare systems in many countries [15]. Following discharge from surgical wards, patients frequently find themselves in an information vacuum regarding available rehabilitation services [16]. Primary care physicians typically have knowledge of only a handful of rehabilitation facilities within their region, critically limiting their ability to effectively direct patient referrals [17]. This results in the systematic violation of two foundational principles of rehabilitation—early initiation and continuity of care—with well-documented adverse consequences for THA outcomes [10].

The healthcare system of Kazakhstan offers a compelling illustration of this problem. A critical imbalance between the supply of and demand for rehabilitation services has emerged, whereby leading national centers performing thousands of THA procedures annually are able to accommodate only a small fraction of patients requiring post-operative rehabilitation, generating waiting lists that extend several months [18,19]. According to the Social Health Insurance Fund, more than 5.9 million deficiencies in medical care provision were identified in 2024 alone, including more than 43,300 documented cases of services billed but never delivered [20,21]. These findings indicate substantial challenges in service monitoring and delivery oversight.

The digitalization of healthcare opens fundamentally new avenues for addressing these challenges [22,23]. Geographic information systems (GIS) have been successfully applied across multiple domains of medicine to analyze the spatial distribution of healthcare resources, optimize patient routing, and improve service accessibility [24]. A rehabilitation coordination platform incorporating GIS functionality has the potential to transform the organization of rehabilitation care by providing transparent, real-time information on available services, actual facility capacity, and access pathways [25,26]. Such a platform could reduce waiting times at overburdened centers, improve utilization of regional facilities, and create objective datasets for quality monitoring and fraud prevention [27,28,29,30].

This study aimed to evaluate the effectiveness of a GIS-integrated mobile platform for coordinating early-stage rehabilitation after THA (Health-GIS) compared to standard referral practice in Kazakhstan. The primary outcomes were time from surgical discharge to the commencement of second-stage rehabilitation—referring, within Kazakhstan’s three-tiered rehabilitation system, to specialized inpatient or residential care following acute surgical discharge—and health-related quality of life as measured by the SF-12 questionnaire. Secondary outcomes included functional recovery assessed by the Harris Hip Score, pain intensity evaluated by the Visual Analogue Scale (VAS), platform usability assessed by the System Usability Scale (SUS), and quality-adjusted life years (QALYs) derived from SF-12 scores over a 12-month follow-up period.

## 2. Materials and Methods

### 2.1. Patient and Public Involvement

No patient or public involvement was sought or required for this study, as it evaluated a digital coordination tool rather than a patient-directed therapeutic intervention.

### 2.2. Study Population and Data Selection

This study was conducted in accordance with the ethical standards of the Declaration of Helsinki and adhered to CONSORT guidelines for reporting randomized controlled trials [31,32]. The protocol received approval from the Local Bioethics Committee of NCJSC Astana Medical University (Protocol No. 11, 26 November 2024) and was subsequently registered on ClinicalTrials.gov (NCT07201116, 23 September 2025). The authors acknowledge that this constitutes retrospective registration. At the time of study initiation, prospective international registration was not a statutory requirement under the applicable regulatory framework in Kazakhstan. The study protocol—including all eligibility criteria, primary and secondary outcomes, randomization procedures, and statistical analysis plan—was established prior to the commencement of recruitment and was not modified at any point after data collection began. The originally planned cost-effectiveness analysis—encompassing the incremental cost-effectiveness ratio (ICER), direct medical costs, and direct non-medical costs—was removed from the outcome list and will be reported in a separate publication dedicated to economic evaluation. Written informed consent was obtained from all participants prior to enrollment.

Adult inpatients aged 18 years or older were recruited at the National Research Oncology Center LLC, Astana, Kazakhstan—a facility that performs elective orthopedic procedures, including primary THA, alongside its core oncological services. Eligibility criteria required that participants: (1) had completed primary total hip arthroplasty within the preceding 7 days; (2) demonstrated basic literacy in Russian or Kazakh, the languages supported by the application; (3) had access to a smartphone or tablet device with internet connectivity; and (4) provided written informed consent to participate. Exclusion criteria comprised severe cognitive or visual impairment, concurrent participation in another clinical trial with potential to interfere with study outcomes, intraoperative or postoperative complications necessitating extended hospitalization following THA, medical contraindications to rehabilitation, planned contralateral THA within 12 months, and voluntary withdrawal following protocol explanation. The 7-day eligibility window was specified to ensure that platform training could be completed during hospitalization, enabling experimental group participants to identify and confirm a rehabilitation placement prior to discharge. Participants received no financial compensation and were free to withdraw at any time without consequence. No specific eligibility criteria were applied to rehabilitation centers participating in the platform; all centers were pre-registered within the Health-GIS platform prior to trial commencement.

### 2.3. Study Design and Randomization

This was a two-arm, parallel-group, superiority randomized controlled trial with a 1:1 allocation ratio. A total of 142 participants were randomized into two groups (control: *n* = 71; experimental: *n* = 71) using simple randomization with a computer-generated sequence prepared by an independent statistician using a validated online randomization tool [33]. Allocation concealment was ensured through a centralized randomization system maintained by the independent statistician; the allocation sequence was inaccessible to personnel responsible for enrolling participants. Recruitment took place from 28 November 2024 to 19 December 2025. Given the nature of the intervention, blinding of participants was not feasible; however, care providers, investigators, and outcome assessors remained blinded to group allocation throughout the study.

### 2.4. Interventions

Participants were randomized to one of two approaches to post-operative rehabilitation coordination following THA.

The experimental group received access to the Health-GIS Rehabilitation Coordination Platform—a specialized mobile application integrating Geographic Information System (GIS) components to facilitate rehabilitation service coordination [34]. Technical infrastructure included Flutter, Dart, Flutterflow for interface creation and Firebase for data storage. During hospitalization, patients submitted rehabilitation requests through the platform, specifying their location, preferred timing, and specific rehabilitation needs. Registered rehabilitation centers received these requests and could respond with counter-proposals detailing available appointment slots, services offered, and estimated costs. The platform provided real-time visualization of available rehabilitation facilities on an interactive map, displaying facility ratings, service offerings, current capacity, and estimated waiting times. Patients received automated notifications regarding new proposals and could compare options based on proximity, service quality ratings, and availability. Technical support was accessible via in-app messaging and a dedicated helpline during business hours.

The control group received the standard rehabilitation referral process. Upon discharge, patients were provided with written recommendations advising rehabilitation as part of their post-operative care plan, but no specific facilities were identified or contact information supplied by the surgical team. Patients were instructed to contact their assigned primary care physician at their local polyclinic for further referral coordination. The primary care physician assumed responsibility for identifying available rehabilitation facilities, generating referrals, and coordinating the rehabilitation timeline based on their knowledge of local resources and current availability. This represents the routine clinical pathway typically followed by patients following THA in Kazakhstan.

Both groups received the same discharge documents and general practitioner (GP) referral instructions, with the GP retaining authority to request specialist clearance when needed. In the experimental group, patients attended the GP appointment with a facility already selected, an admission date confirmed via Health-GIS, and a list of required pre-admission tests generated by the facility. Also, both groups received standard post-operative pharmacological management in accordance with institutional protocols, with no significant differences between groups in this regard.

All participating facilities were inpatient rehabilitation departments operating under unified national rehabilitation regulations [35,36], which define minimum standards for programme content, physiotherapy procedures, and equipment. Based on the standard rehabilitation programmes typically delivered at registered inpatient facilities in Kazakhstan, patients generally receive a combination of active individual kinesiotherapy of the lower extremity, group kinesiotherapy, continuous passive motion therapy using a CPM device, and individual cardiorespiratory training incorporating a stationary bicycle, resistance bands for lower limb strengthening, an active flexion–extension device with load, and Swiss ball exercises. Physiotherapy modalities typically included magnetic therapy and laser therapy sessions. However, specific intensity and duration of individual programmes were not directly monitored in this study, and day-to-day implementation may vary across facilities. The groups differed solely in their referral routing mechanism.

### 2.5. Workflow and User Interaction Within the Application

**Figure 1:**
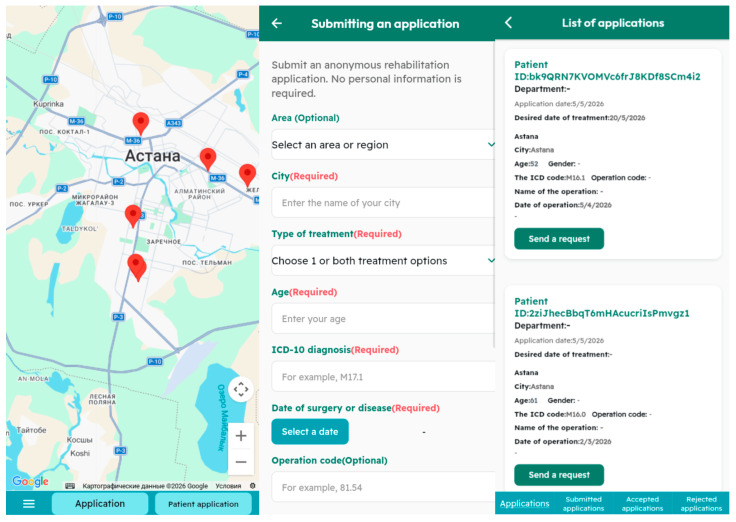
Application user interface screens.

The application implements a bidirectional workflow connecting patients with rehabilitation centers across four sequential stages, as illustrated in Figure 1.

Center discovery. Upon login, patients are presented with an interactive map displaying available rehabilitation centers in their region. Selecting a center provides access to detailed facility information, including departments, physicians, available treatment modalities, and patient reviews.

Anonymous request submission. Patients may submit an anonymous treatment request by specifying the required department, along with fields like ICD code, operation code, desired rehabilitation date, and comments. A privacy notice confirms that no personal data is collected. The completed request is distributed to all registered centers in the system.

Review and selection. Rehabilitation centers receive incoming requests in a dedicated queue displaying anonymous patient data.

Pre-admission coordination. Following acceptance by a center, the patient is notified and may review the responding facility’s profile. Upon selecting a preferred center, both parties initiate direct communication through the integrated messaging system to confirm hospitalization conditions, required laboratory tests, documentation, and proposed admission dates.

### 2.6. Outcomes

The study specified two primary outcomes: (1) Time to Rehabilitation Initiation, defined as the number of days elapsed from discharge from the surgical hospital to commencement of second-stage rehabilitation services; and (2) SF-12 Quality of Life Score, assessed using the 12-Item Short Form Health Survey, yielding a Physical Component Summary (PCS) and Mental Component Summary (MCS), each scored from 0 to 100, with higher scores indicating better health status [37]. The SF-12 was administered at baseline, 6 months, and 12 months from enrollment.

Secondary outcomes included: (3) the Harris Hip Score, a composite measure of hip joint function, pain, and mobility scored from 0 to 100, with higher scores indicating superior function and lesser disability, assessed at baseline [38], 6 weeks, 6 months, and 12 months; (4) the Visual Analogue Scale (VAS) Pain Score [39], measuring pain intensity on a 0–10 scale, where 0 denotes no pain, and 10 denotes the worst imaginable pain, assessed at baseline, 6 weeks, 6 months, and 12 months; (5) the System Usability Scale (SUS) Score [40,41,42], evaluating usability of the Health-GIS platform among both patients and healthcare facilities on a scale of 0 to 100, with higher scores reflecting greater usability and user satisfaction, assessed at 3 and 6 months from enrollment; and (6) Quality-Adjusted Life Years (QALYs) [43], calculated from SF-6D utility scores derived from SF-12 responses using the trapezoidal rule over the 12-month study period [44,45], with higher values representing more time spent in better health states, assessed at 12 months. Serious adverse events attributable to the surgical procedure—including prosthetic dislocation, deep infection requiring revision surgery, unplanned hospitalization, and death—were monitored throughout the 12-month follow-up and recorded at each scheduled assessment visit.

### 2.7. Statistical Analysis

All statistical analyses were performed using IBM SPSS Statistics (version 27.0.1.0). Normality of data distribution was assessed using the Kolmogorov–Smirnov test. Normally distributed quantitative variables are reported as mean ± standard deviation (M ± SD) with 95% confidence intervals (95% CI); non-normally distributed data are expressed as median and interquartile range (Me [Q1–Q3]). Between-group comparisons of demographic and baseline characteristics were conducted using the independent-samples *t*-test for normally distributed variables and the Mann–Whitney U test for non-normally distributed variables. Categorical variables were compared using Pearson’s chi-square test. All statistical tests were two-tailed, with a significance threshold of *p* < 0.05.

No interim analyses were planned or conducted, and no stopping rules were pre-specified. Analyses were performed on a modified intention-to-treat basis, encompassing all randomized participants who received the allocated intervention and completed at least one post-baseline assessment. Missing data arising from participant withdrawal were handled using a complete case approach; participants who declined further follow-up and provided no outcome data after the baseline visit were excluded from all analyses. No imputation of missing values was performed. No subgroup analyses were pre-specified.

### 2.8. Sample Size Calculation

Sample size was calculated a priori using G*Power (version 3.1.9.7), based on the “difference between two independent means” test. The following parameters were applied: two-tailed test, Cohen’s d = 0.5 (medium effect size), α = 0.05, power = 0.80, and a 1:1 allocation ratio. This effect size was considered both clinically meaningful and appropriately conservative for an intervention designed to improve outcomes over standard practice [46]. The analysis yielded a required sample of 64 participants per group (128 total). To account for an anticipated attrition rate of approximately 10% over the 12-month follow-up period, the final recruitment target was set at 142 participants (71 per group).

## 3. Results

### 3.1. Trial Completion and Participant Flow

**Figure 2:**
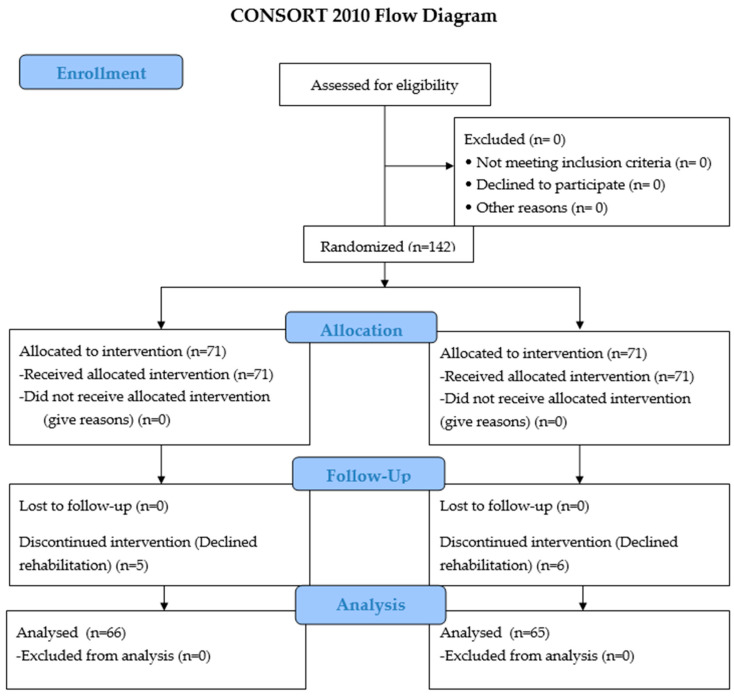
CONSORT flow diagram illustrating participant progression through the study.

The trial was completed as planned following attainment of the pre-specified sample size. Participant flow through each stage of the trial is illustrated in Figure 2. Of 158 THA candidates evaluated during the recruitment period, 16 were identified as ineligible for surgery due to severe cognitive impairment or major medical contraindications and did not proceed to the operative stage; these individuals were never admitted to the post-operative ward and were therefore not formally assessed for study participation. The remaining 142 consecutive post-operative patients were formally assessed for study eligibility, and all 142 met all inclusion criteria and none of the exclusion criteria, yielding zero study-level screen failures. The final modified intention-to-treat analysis included 66 participants in the experimental group and 65 in the control group. A post hoc power calculation confirmed that the final analysed sample of 131 participants (*n* = 66 experimental, *n* = 65 control) retained adequate statistical power of 0.81 (G*Power 3.1.9.7; two-tailed *t*-test, Cohen’s d = 0.5, α = 0.05), exceeding the pre-specified threshold of 0.80.

### 3.2. Baseline Characteristics

Baseline sociodemographic characteristics were well balanced between groups, with no statistically significant differences in age, BMI, or sex distribution (Table 1). Groups were balanced at baseline by randomization; no additional matching was performed. Baseline comparability was confirmed statistically (Table 1). Enrolled participants ranged in age from 44 to 78 years.

### 3.3. Time to Rehabilitation Initiation

Participants in the experimental group began rehabilitation significantly earlier than those in the control group (median 43 vs. 59 days post-discharge; *p* = 0.021), representing a 16-day earlier start to rehabilitation.

### 3.4. Health-Related Quality of Life (SF-12)

#### 3.4.1. Physical Component Summary (PCS)

PCS scores were comparable between groups at baseline (*p* = 0.199) and at 6 months (*p* = 0.190), with both groups demonstrating significant within-group improvement over this period (Table 2). By 12 months, the experimental group maintained superior physical functioning while the control group declined, yielding a statistically significant between-group difference (*p* < 0.001). Over the full 12-month period, the experimental group achieved significant improvement from baseline (*p* < 0.001), whereas the corresponding change in the control group did not reach statistical significance (*p* = 0.181).

#### 3.4.2. Mental Component Summary (MCS)

MCS scores were comparable between groups at all time points (Table 3). Over the full 12-month period, both groups achieved significant within-group improvements in mental health functioning, with no significant between-group difference.

### 3.5. Pain Assessment (VAS)

Both groups exhibited progressive pain reduction throughout the study period, with highly significant within-group improvements from baseline to 12 months (*p* < 0.001). No statistically significant between-group differences were observed at any time point (Table 4).

### 3.6. Hip Function (Harris Hip Score)

Both groups demonstrated progressive improvement in Harris Hip Scores throughout follow-up (Table 5). The experimental group showed significantly superior scores at 6 weeks (*p* < 0.001) and 6 months (*p* = 0.009). By 12 months, the between-group difference no longer reached statistical significance (*p* = 0.068), suggesting convergence of functional trajectories over time.

### 3.7. Quality-Adjusted Life Years (QALYs)

The experimental group demonstrated significantly higher 12-month QALY scores compared to the control group (*p* = 0.008) (Table 6).

### 3.8. Platform Usability (System Usability Scale)

#### 3.8.1. Physician Assessment

Platform usability was evaluated by four physicians using the SUS at 3 and 6 months following implementation. At 3 months, mean individual SUS scores were 75.0, 75.0, 85.0, and 77.5, yielding an overall mean of 78.1. Physicians expressed strong agreement on several positive dimensions: all indicated a desire to use the system frequently (mean item score: 4.25), found it easy to use (4.25), considered its functions well integrated (4.00), believed most people would learn to use it quickly (4.00), and felt confident using it (4.25). On reverse-scored items, physicians consistently reported that the system was not unnecessarily complex (mean: 2.00), did not require technical support to operate (1.75), was not inconsistent in its behavior (2.00), was not cumbersome to use (2.00), and required minimal prior learning (1.75).

By 6 months, usability ratings had improved, with mean individual scores of 87.5, 85.0, 92.5, and 82.5, yielding an overall mean of 86.9. All positively framed items received higher ratings than at 3 months (mean item scores of 4.5 across frequency of use, ease of use, functional integration, ease of learning, and confidence). Reverse-scored items similarly reflected sustained improvement, with physicians reporting minimal complexity (1.75), low need for technical support (1.50), good consistency (1.50), ease of use (1.50), and a minimal learning curve (1.50).

#### 3.8.2. Patient Assessment

Platform usability was additionally evaluated by 20 patients using the SUS. Individual scores ranged from 57.5 to 92.5, with a mean of 71.4. The majority of patients (*n* = 12, 60%) achieved scores of 75 or above, indicative of good to excellent usability. Five patients (25%) scored between 60.0 and 72.5, suggesting marginally acceptable usability with scope for improvement, while three patients (15%) scored 57.5, reflecting the lower threshold of marginally acceptable usability.

### 3.9. Adverse Events

No serious adverse events related to the study intervention were observed in either group throughout the 12-month follow-up period.

## 4. Discussion

The most notable finding of this randomized controlled trial was that the Health-GIS platform reduced the time to initiation of second-stage rehabilitation after THA by 16 days compared with the standard referral pathway (median 43 vs. 59 days; *p* = 0.021). This effect is clinically relevant because it occurred during the early post-operative recovery window, when restoration of mobility, strengthening, gait correction, and prevention of maladaptive movement patterns are central objectives of rehabilitation [8,9,10,11,12,13,47]. Accordingly, the functional benefits observed in this trial are best understood as a consequence of reduced time-to-rehabilitation rather than of any modification to the physical therapy programme itself [8,12].

The improvement in the SF-12 Physical Component Summary (PCS) supports the clinical relevance of earlier rehabilitation initiation. At 12 months, PCS was higher in the experimental group than in the control group (48.21 vs. 42.84; *p* < 0.001), producing a between-group difference of 5.37 points. This exceeds published estimates of clinically important change for SF-12 PCS in orthopaedic populations, where MCID and MIC values of approximately 1.8 and 2.7 points, respectively, have been reported [48]. The PCS result may therefore be interpreted not only as statistically significant, but also as clinically meaningful. In contrast, SF-12 Mental Component Summary (MCS) scores did not differ significantly between groups at 12 months (46.96 vs. 47.05; *p* = 0.669), indicating that the platform primarily influenced physical recovery and access coordination rather than broader psychological well-being. This is consistent with evidence that psychosocial outcomes after THA are governed primarily by variables such as anxiety, sense of coherence, and social support, none of which are directly influenced by the timing of rehabilitation initiation [49].

The QALY result requires a more cautious interpretation. The experimental group had a statistically higher 12-month QALY value than the control group (0.74 ± 0.04 vs. 0.72 ± 0.04; *p* = 0.008), but the absolute between-group difference was 0.02. This is below the commonly reported minimally important differences for generic utility-based measures, including approximately 0.041 for SF-6D, 0.074 for EQ-5D, and about 0.03 for generic utility measures [50]. Accordingly, the QALY gain should be described as statistically significant but clinically modest. This distinction is important because small utility gains may still matter at the population level, particularly if achieved through scalable coordination tools, but should not be overstated at the individual-patient level.

Functional recovery assessed by the Harris Hip Score showed an early advantage for the experimental group at 6 weeks (66.96 vs. 56.30; *p* < 0.001) and 6 months (84.03 vs. 80.03; *p* = 0.009), with convergence by 12 months (87.03 vs. 84.03; *p* = 0.068). This pattern suggests that earlier rehabilitation accelerates recovery without necessarily changing the final functional plateau at one year, consistent with evidence that patient-reported outcomes after THA often plateau by 6–12 months [51].

The absence of between-group differences in VAS pain scores is clinically plausible. The Health-GIS intervention did not include a pain self-management module, medication titration, or remote symptom monitoring. Pain reduction occurred similarly in both groups and was likely driven primarily by surgery, standard post-operative pharmacological care, natural recovery, and individual patient-related factors. Systematic review evidence indicates that persistent pain after THA is influenced by preoperative pain, sex, comorbidities, and other patient- and procedure-related variables [52].

Platform usability assessments yielded encouraging results. Healthcare providers rated the system highly, with a mean SUS score of 78.1 at 3 months and rising to 86.9 at 6 months, consistent with an “excellent” classification and reflecting progressive user adaptation over time [41]. Among patients, the mean SUS score was 71.4, approaching the threshold for “good” usability, with 60% of patients scoring 75 or above.

### 4.1. Strengths and Limitations

The main strengths of this study include its randomized controlled design, a priori sample size calculation, 12-month follow-up, blinded outcome assessment, and use of validated instruments, including the SF-12, Harris Hip Score, VAS, SUS, and SF-6D-derived QALYs. The final analysed sample of 131 participants exceeded the minimum required sample of 128, preserving adequate statistical power despite attrition.

Several limitations should be acknowledged. First, participant blinding was not feasible because the intervention required active use of the platform. This may have introduced performance bias, as patients using a new digital tool may have been more engaged with rehabilitation planning independently of any objective system benefit.

Second, surgical care was provided at a single institution in Kazakhstan, while rehabilitation was delivered across multiple inpatient departments within the same city. Although this reflects real-world conditions, it limits external validity, as results may differ in healthcare systems with other referral architectures, insurance mechanisms, rehabilitation capacity, or digital infrastructure.

Third, eligibility required smartphone or tablet access, internet connectivity, and basic digital literacy. This constitutes a selection bias and may systematically exclude older adults with limited digital skills, socioeconomically disadvantaged patients, and residents of remote areas with unreliable internet access. Although socioeconomic status was not directly measured, several contextual factors may have reduced its influence on rehabilitation access in this study. Internet penetration in Kazakhstan is high, with 95.6% of individuals aged 16–74 years reported to use the Internet [53]. In addition, all participants who underwent THA and were eligible for post-operative rehabilitation had compulsory social health insurance coverage, allowing access to second-stage rehabilitation without direct patient payment; national insurance coverage exceeds 90% of the population [54]. These factors suggest that socioeconomic status was unlikely to be the primary determinant of whether patients could find and receive rehabilitation. Nevertheless, residual socioeconomic influences cannot be excluded, particularly regarding digital confidence, transport availability, family support, and the ability to navigate administrative procedures—none of which were directly measured in this study. The findings, therefore, apply most directly to digitally enabled post-THA patients and should not be generalized uncritically to all rehabilitation candidates.

Fourth, usability testing was limited to four physicians and twenty patients. Although SUS scores were acceptable among patients and high among clinicians, this sample is too small to characterize usability across broader patient groups. The lower and more variable patient SUS scores indicate that simplified interfaces, assisted onboarding, and telephone or polyclinic-based support pathways may be necessary before large-scale implementation.

Finally, this study did not include a formal economic evaluation. The analysis included QALY estimation but did not capture platform development costs, maintenance costs, staff time, direct rehabilitation costs, or incremental cost-effectiveness ratios. The present findings therefore support clinical and organizational effectiveness but do not yet establish cost-effectiveness. However, it should be noted that the platform was developed by the study authors without commercial costs, and both groups received identical rehabilitation programmes under the same national tariffs. These factors suggest that the incremental cost of the intervention relative to standard referral practice is likely to be modest. A dedicated economic analysis is nonetheless required before policy-level scaling decisions can be made.

### 4.2. Comparison with Related Studies and Mechanistic Interpretation

The present findings align with the broader digital health literature showing that integrated digital platforms can improve care coordination after joint arthroplasty. Colomina et al. evaluated an mHealth-enabled integrated care platform for patients undergoing hip or knee arthroplasty and reported reductions in unplanned healthcare utilization and favourable cost-utility results [55]. However, that intervention supported integrated care and self-management rather than GIS-based selection of rehabilitation providers. The present study adds a distinct contribution by focusing on access coordination and time to rehabilitation initiation as primary outcomes.

GIS has previously been used to evaluate geographic access to specialized healthcare services, including radiotherapy planning, and can reveal spatial inequities not visible in aggregate service statistics [56]. The Health-GIS platform operationalizes these principles at the patient-facing level by showing available departments, enabling request submission, and facilitating direct communication between patients and providers.

The likely mechanism of benefit is organizational rather than biological. Traditional referral systems depend on incomplete knowledge of available facilities, sequential communication, and administrative friction. Platform-based coordination may reduce these transaction costs by consolidating facility discovery, availability confirmation, and pre-admission communication in a single digital workflow [55,57]. It may also redistribute patient flows away from overburdened departments toward facilities with available capacity, thereby reducing waiting time without requiring immediate expansion of infrastructure.

### 4.3. Implications and Future Research

For clinical practice, the results support the integration of rehabilitation coordination tools into discharge planning after THA. The platform should be viewed as an adjunct to standard referral rather than as a replacement for clinical assessment. Its practical value lies in equipping the patient and GP with concrete information: selected rehabilitation department, proposed admission date, available capacity, and required pre-admission tests.

For health systems, the findings suggest that delays in post-operative rehabilitation are partly informational and organizational. Digital documentation of requests, responses, waiting times, and admissions may also improve transparency of service delivery and support monitoring of rehabilitation access, particularly in systems where a mismatch between declared and actually delivered services is a policy concern [20,21,30].

Future iterations of the platform should incorporate alternative access pathways—including web-based interfaces, telephone-assisted navigation, and simplified interfaces—to extend reach to patients without smartphones or with limited digital proficiency. Future research should prioritize multicenter trials across different regions, inclusion of patients with limited digital literacy through assisted-access models, detailed measurement of rehabilitation dose and adherence, and formal cost-effectiveness analysis from both healthcare system and societal perspectives. Longer follow-up is also needed to determine whether earlier rehabilitation initiation affects sustained function, revision risk, and long-term quality of life.

## 5. Conclusions

This randomized controlled trial demonstrated that a GIS-integrated rehabilitation coordination platform significantly reduced time to second-stage rehabilitation initiation by 16 days and improved physical functioning and health-related quality of life following total hip arthroplasty. These findings suggest that delays in post-operative rehabilitation are partly informational and organizational, and therefore amenable to digital solutions. The platform demonstrated acceptable usability among both clinicians and patients and is unlikely to impose substantial additional costs given identical rehabilitation programmes across groups. Multi-center trials and formal cost-effectiveness analyses are needed to establish generalizability and support scaling decisions.

## Figures and Tables

**Table 1:** Baseline characteristics of study participants.

Characteristics	Participants (*n* = 131)	Experimental Group (*n* = 66)	Control Group (*n* = 65)	*p*-Value
Socio-demographic characteristics				
Age, years (Mean ± SD)	64.99 ± 7.08	65.73 ± 6.93	64.25 ± 7.21	0.233
BMI, kg/m^2^ (Median [IQR])	27.20 [23.70–30.80]	26.95 [24.40–30.58]	27.60 [23.50–31.05]	0.652
Gender, *n* (%)				
Male	80 (61.1%)	43 (65.2%)	37 (56.9%)	0.334
Female	51 (38.9%)	23 (34.8%)	28 (43.1%)	

**Table 2:** SF-12 Physical Component Summary (PCS) scores by group and time point.

Time Point	Intervention Group (*n* = 66)	Control Group (*n* = 65)	*p*-Value (Between Groups)
Baseline, Median [IQR]	39.91 [35.43–44.38]	41.36 [36.83–46.81]	0.199
6 months, Median [IQR]	48.00 [42.85–51.42]	45.58 [41.87–50.80]	0.190
12 months, Median [IQR]	48.21 [44.25–52.25]	42.84 [37.24–49.53]	<0.001
Within-group changes (*p*-value)			
0 → 6 months	<0.001	0.002	
6 → 12 months	0.581	0.122	
0 → 12 months	<0.001	0.181	

**Table 3:** SF-12 Mental Component Summary (MCS) scores by group and time point.

Time Point	Intervention Group (*n* = 66)	Control Group (*n* = 65)	*p*-Value (Between Groups)
Baseline, Median [IQR]	45.87 [39.11–49.75]	44.75 [38.27–48.46]	0.237
6 months, Median [IQR]	46.68 [43.33–51.65]	46.39 [42.91–50.46]	0.861
12 months, Median [IQR]	46.96 [43.45–52.75]	47.05 [44.13–51.13]	0.669
Within-group changes (*p*-value)			
0 → 6 months	0.107	0.003	
6 → 12 months	0.394	0.751	
0 → 12 months	0.004	<0.001	

**Table 4:** Visual Analogue Scale (VAS) by group and time point.

Time Point	Intervention Group (*n* = 66)	Control Group (*n* = 65)	*p*-Value (Between Groups)
Baseline, Median [IQR]	5 [4–7]	5 [4–7]	0.814
6 weeks, Median [IQR]	3 [2–4]	3 [2–5]	0.336
6 months, Median [IQR]	2 [2–3]	2 [2–3]	0.066
12 months, Median [IQR]	1 [1–2]	1 [1–2]	0.105
Within-group changes (*p*-value)			
0 → 6 weeks	<0.001	<0.001	
6 weeks → 6 months	<0.001	<0.001	
6 months → 12 months	<0.001	<0.001	
0 → 12 months	<0.001	<0.001	

**Table 5:** Harris Hip Scores (HHSs) by group and time point.

Time Point	Intervention Group (*n* = 66)	Control Group (*n* = 65)	*p*-Value (Between Groups)
Baseline, Median [IQR]	46.64 [35.93–56.77]	46.35 [33.05–56.17]	0.816
6 weeks, Median [IQR]	66.96 [57.39–79.49]	56.30 [47.49–65.27]	<0.001
6 months, Median [IQR]	84.03 [74.03–89.03]	80.03 [67.53–86.03]	0.009
12 months, Median [IQR]	87.03 [82.03–93.03]	84.03 [77.03–90.03]	0.068
Within-group changes (*p*-value)			
0 → 6 weeks	<0.001	<0.001	
6 weeks → 6 months	<0.001	<0.001	
6 months → 12 months	<0.001	<0.001	
0 → 12 months	<0.001	<0.001	

**Table 6:** Comparison of 12-month quality-adjusted life years (QALYs) between intervention and control groups.

Outcome Measure	Intervention Group	95% CI	Control Group	95% CI	*p*-Value
12-month QALY	0.74 ± 0.04	0.73–0.75	0.72 ± 0.04	0.71–0.73	0.008

## Data Availability

Data are contained within the Supplementary Material.

## Supplementary Materials

The following supporting information can be downloaded at: https://www.mdpi.com/article/10.3390/ijerph23060751/s1, Table S1: Patient-level outcome data by group and time point.

## Abbreviations

The following abbreviations are used in this manuscript:

BMI	Body mass index
CI	Confidence interval
GIS	Geographic information system
IQR	Interquartile range
MCS	Mental Component Summary
PCS	Physical Component Summary
QALY	Quality-adjusted life year
RCT	Randomized controlled trial
SD	Standard deviation
SF-6D	Short Form Six-Dimension
SF-12	12-Item Short Form Health Survey
SPSS	Statistical Package for the Social Sciences
SUS	System Usability Scale
THA	Total hip arthroplasty
VAS	Visual Analogue Scale
